# Effects of Dietary Protein Quantity, Source, and Type on Plasma Lipids and Lipoproteins and Their Roles in Dyslipidemia Management in Humans

**DOI:** 10.3390/nu18132207

**Published:** 2026-07-07

**Authors:** Kevin C. Maki, Mary R. Dicklin, Carol F. Kirkpatrick, Orsolya M. Palacios

**Affiliations:** 1 Midwest Biomedical Research, Addison, IL 60101, USA; mdicklin@mbclinicalresearch.com (M.R.D.); opalacios@mbclinicalresearch.com (O.M.P.); 2 Department of Applied Health Science, Indiana University School of Public Health-Bloomington, Bloomington, IN 47405, USA; 3 Kasiska Division of Health Sciences, Idaho State University, Pocatello, ID 83209, USA

**Keywords:** dietary protein, dietary guidelines, animal-based protein, plant-based protein, lipoprotein lipids, cardiometabolic disease

## Abstract

Evidence from clinical trials indicates that dietary protein plays an important and often underappreciated role in lipoprotein lipid metabolism. For this narrative review, literature searches were conducted in the PubMed and Cochrane Central Register of Controlled Trials databases for articles describing randomized controlled trials (RCTs) and systematic reviews and meta-analyses of RCTs, as well as dietary guidelines and dyslipidemia management recommendations, using search terms for protein quantity, source (e.g., animal- and plant-based), and type (e.g., dairy, meat, soy, and nuts) and effects on lipids and lipoproteins in humans. Findings indicated that dietary intakes of both animal-based and plant-based proteins, when replacing refined carbohydrates or saturated fatty acids, lower circulating concentrations of atherogenic lipoproteins. Protein from plant sources appears to produce a somewhat larger effect on lipoprotein lipid concentrations than protein from animal sources. Individual amino acids (e.g., branched-chain amino acids), protein food fractions (e.g., whey), and food-derived peptides may independently impact lipoprotein lipid metabolism. Beyond the effect of replacing one macronutrient for another, the biochemical pathways responsible for the effects of dietary protein on lipoprotein lipid metabolism in humans have not been fully defined. The importance of dietary protein in a healthy diet is emphasized in recent dietary recommendations for the general population and for individuals with dyslipidemias. Additional research is warranted to determine the amount of dietary protein and the best balance of food source(s) to optimize its benefits on lipoprotein lipid concentrations, as well as the mechanisms for these effects.

## 1. Introduction

Atherosclerotic cardiovascular disease (ASCVD) is one of the leading causes of death worldwide [1]. Dyslipidemias, particularly those associated with an increase in the circulating burden of atherogenic apolipoprotein (Apo) B-containing lipoproteins, such as hypercholesterolemia (elevated total cholesterol and low-density lipoprotein cholesterol [LDL-C]), hypertriglyceridemia (elevated triglycerides [TG]), and mixed dyslipidemia (elevated LDL-C and TG), are well-established ASCVD risk factors [1,2,3]. LDL-C and non-high-density lipoprotein cholesterol (non-HDL-C), which is calculated as the concentration of total cholesterol minus the concentration of high-density lipoprotein cholesterol (HDL-C) and is a comprehensive measurement of cholesterol carried by all Apo B-containing lipoproteins, are the targets for therapies to lower ASCVD risk [4]. Changes in both have been shown to predict changes in risk, and these relationships are believed to reflect a causal association of the burden of atherogenic lipoprotein particles with the initiation and progression of ASCVD. Changes in HDL-C are of uncertain clinical relevance [5]. The TG concentration correlates strongly with the level of very low-density lipoprotein cholesterol (VLDL-C). Therefore, changes in non-HDL-C reflect the net effect of changes in LDL-C and TG, since the two major components of non-HDL-C are LDL-C and VLDL-C. Evidence indicates that non-HDL-C is a stronger correlate of Apo B, and both non-HDL-C and Apo B are superior to LDL-C as predictors of ASCVD risk [6].

Age, sex, genetics, and baseline lipid phenotype are key determinants of blood lipid levels, but lifestyle behaviors, including dietary patterns, are also important contributors to lipoprotein lipid concentrations [3,7,8,9,10,11]. Each dietary macronutrient influences cardiovascular health, in part due to impacts on circulating lipoprotein lipids as well as other cardiometabolic biomarkers [12]. The effects of dietary fat and carbohydrates on lipoprotein lipids are well described [13]. In brief, C12:0-C16:0 saturated fatty acids (SFAs) raise LDL-C; C18:0 (stearic acid) has a mainly neutral effect; omega-6 polyunsaturated fatty acids (PUFA; primarily linoleic acid) and omega-9 monounsaturated fatty acids (MUFA; primarily oleic acid) lower LDL-C; industrial *trans* fatty acids increase LDL-C; and dietary carbohydrates, particularly refined carbohydrates and simple sugars, increase TG and VLDL-C concentrations [13]. Accordingly, dietary guidance for ASCVD risk reduction has emphasized changes in the quantity and types of dietary fats and carbohydrates but has not generally focused on the potential role protein may play in lipoprotein metabolism.

The purpose of this review is to raise awareness among clinicians of the effects of dietary protein on lipoprotein lipids and to stimulate further research on the clinical implications and mechanisms underlying these responses. The originality of this review lies in its integrated consideration of protein quantity, source, and type within the context of dyslipidemia management. Specifically, this review emphasizes evidence from randomized controlled trials (RCTs) evaluating the effects of dietary proteins on lipoprotein lipids, focusing primarily on LDL-C, non-HDL-C, and Apo B for the reasons noted above. The evidence is also examined to describe the impacts of protein amount, type and source, as well as the potential effects of reducing the macronutrient(s) being displaced by protein. The review also summarizes dietary guidelines and dyslipidemia management recommendations related to protein and provides a brief overview of potential mechanisms relevant to lipoprotein metabolism and ASCVD risk.

## 2. Literature Search

Literature searches were conducted in the PubMed and Cochrane Central Register of Controlled Trials databases for articles in English describing RCTs, systematic reviews and meta-analyses of RCTs, as well as dietary guideline and dyslipidemia management recommendations from national and international organizations using search terms related to dietary protein quantity, protein source (e.g., animal- and plant-based), and protein food type (e.g., dairy, meat, soy, and nuts) and effects on lipids and lipoproteins in humans. Studies of whole protein foods as well as protein isolates (e.g., soy protein isolate, whey, and casein) with varying levels of processing (e.g., unprocessed and processed meats) were considered. Priority was given to studies that were well controlled (e.g., foods were provided) to minimize the potential for confounding from other factors such as dietary fiber, fatty acids, or cholesterol. Studies that reported results for LDL-C and non-HDL-C (or TG if non-HDL-C was not reported) were also prioritized. There was no date filter used in the literature searches, but articles from the past 20 years were of particular interest for this narrative review.

## 3. Protein Recommendations in Dietary Guidelines for the General Population

The U.S. Department of Health and Human Services and the U.S. Department of Agriculture released the 2025–2030 Dietary Guidelines for Americans (DGAs), 10th edition, in January 2026 [14,15]. The DGAs recommend that the general population follow a healthy dietary pattern that emphasizes whole, unprocessed, or minimally processed foods and is high in fiber-rich components, such as fruits, vegetables, whole grains, nuts, and seeds. The updated DGAs also emphasize healthy fats and proteins, including plant-based protein (beans, peas, lentils, legumes/pulses, nuts, seeds, soy) and, depending on the dietary pattern, animal-based protein (fish, seafood, eggs, dairy, poultry, red meat) [14]. A major change in the 2025–2030 DGAs, compared to earlier versions, was an increase in the daily dietary protein recommendation to 1.2–1.6 g/kg of body weight/d, adjusted as needed for individual caloric needs [14], instead of the Recommended Dietary Allowance for protein of 0.8 g/kg body weight/d aimed at preventing deficiency [16]. The Acceptable Macronutrient Distribution Range for protein, i.e., the percent of calories from total daily energy (TDE), is 10–35% for adults, which corresponds to ~1.05–3.67 g protein/kg body weight/d (reference body weights for women and men of 57 kg and 70 kg, respectively) [16]. Additional recommendations include prioritizing nutrient-dense, high-quality protein foods at every meal, eating a variety of animal- and plant-based protein foods, eating meat with no or limited added sugars, refined carbohydrates, starches, or chemical additives, and choosing to bake, broil, roast, stir-fry, or grill rather than deep-frying. There is controversy surrounding several of the recommendations in the 2025–2030 DGAs, including the guidance to consume more protein [17,18,19].

The authors of the Scientific Foundation Report for the 2025–2030 DGAs explained that their rationale for promoting a moderately higher intake of dietary protein than the current average intake of ~1 g/kg of body weight/d was largely based on the potential advantages of higher protein intake for body weight and composition [15,20,21,22]. In a systematic review of 30 RCTs that examined higher-protein diets (1.2–1.6 g/kg body weight), compared with lower-protein diets (0.8–1.0 g/kg), on weight-related outcomes, 68% of the RCTs reported significant improvements in at least one primary weight loss/management outcome (body weight, body fat, fat free/lean mass, body mass index, or waist circumference) with no adverse effects [15]. The durations of these trials were 12 weeks to 2 years, and most were in adults with overweight or obesity during calorie restriction, with the higher intake of protein achieved with animal-source foods [15]. While increasing daily protein intake enhances muscle hypertrophy and strength and preserves lean mass when combined with regular resistance or endurance training [23,24], there are concerns that, without such training, excess protein intake might be harmful [18,25,26].

The authors of a recent commentary examined the evidence for possible harms of higher protein intake and concluded that, in healthy adults, intervention studies in humans do not support increased risks for kidney damage, increased bone loss, or fracture; there is insufficient evidence to rule out potential effects on mortality, but evidence to suggest implausibility; and there is sufficient evidence suggesting plausibility of increased risk for type 2 diabetes mellitus, although alleged harm has not been demonstrated [27]. However, the authors noted that understanding the characteristics of the studies they evaluated is important to put these conclusions in context, including: the dietary interventions evaluated, how the study participants’ actual protein needs were determined, the experimental models utilized, the intermediate disease biomarkers assessed, and the length of the intervention periods. For example, the evidence evaluated for kidney dysfunction was mostly from studies of relatively short duration (≤2 years). Therefore, the possibility of adverse effects with long-term exposure to higher protein intake could not be ruled out [27]. Additional research to further examine potential harms and benefits of higher protein intake is needed.

The World Health Organization (WHO) recommends consuming 10–15% of TDE as protein, stating that this range is generally sufficient to meet the needs of adults, although protein intake higher than 15% may be appropriate during adolescence and for athletes, body builders, and others who are actively building or maintaining substantial muscle mass [28]. Like the DGAs, the WHO states that protein can come from a mix of animal and plant sources but notes that, in some contexts, plant sources of protein may be preferable to decrease disease risk in adults, and animal sources of protein may be preferable for some groups with specific nutrient needs, particularly children and pregnant or lactating women. Most European countries have their own national dietary guidelines that broadly align with the WHO’s recommendations, although differences do exist [29]. For example, food categories in different countries’ recommendations do not consistently include the same food products, e.g., legumes might be categorized with fruits and vegetables, with proteins, or in their own category. Also, some countries describe in their recommendations the potential benefits to the environment of consuming plant-based vs. animal-based proteins [30]. The environmental impact of increasing intake of dietary protein and plant- vs. animal-based proteins is beyond the scope of this manuscript, but readers interested in this topic are referred to a publication by Jennings et al. describing the relationships of five U.S. dietary patterns, and specific food groups within those dietary patterns, with land and water use and greenhouse gas emissions [31].

## 4. Dietary Protein Recommendations for the Management of Dyslipidemias

**Figure 1:**
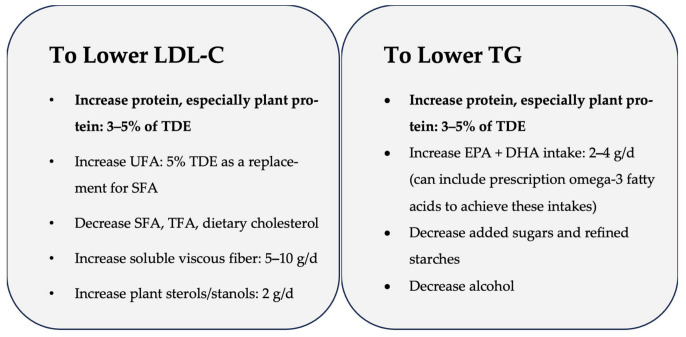
Key components of dietary interventions for the management of dyslipidemias [8]. Abbreviations: DHA, docosahexaenoic acid; EPA, eicosapentaenoic acid; LDL-C, low-density lipoprotein cholesterol; SFA, saturated fatty acids; TDE, total daily energy; TG, triglycerides; TFA, *trans* fatty acids; UFA, unsaturated fatty acids.

Beyond dietary recommendations for the general population, several national and international organizations, including the National Lipid Association (NLA), the American College of Cardiology, the American Heart Association, the European Atherosclerosis Society, and the European Society of Cardiology, provide dietary recommendations for the management of dyslipidemias and/or promotion of optimal cardiovascular health [3,7,8,9,10,11,32,33]. These recommendations generally suggest that individuals with dyslipidemias and/or at risk for ASCVD follow the healthy eating patterns recommended for the general population, which emphasize fruits, vegetables, nuts, seeds, legumes, whole grains, healthy fats and proteins, with added focus on reducing dietary factors that increase LDL-C and TG and increasing dietary factors that lower LDL-C and TG (Figure 1) [8].

The average U.S. diet has higher than recommended levels of SFA, sodium, refined starches, and added sugars. In fact, approximately 50% of TDE comes from carbohydrates in the average U.S. diet, and the majority is derived from refined starches and added sugars [34]. For individuals with elevated LDL-C, the aim is to lower atherogenic, Apo B-containing particles. This is accomplished by: (1) replacing foods high in SFA, dietary cholesterol, and *trans* fatty acids (although industrial *trans*-fats have been mainly eliminated from the U.S. food supply), with foods rich in unsaturated fatty acids (UFA), including MUFA and PUFA; (2) partially replacing SFA, refined carbohydrates, and added sugars with a combination of UFA and protein, especially plant-based protein; and (3) increasing consumption of foods rich in viscous soluble fibers and plant sterols/stanols [8,10]. For individuals with elevated TG, the aim is to lower TG-rich lipoproteins by limiting foods and beverages with refined carbohydrates, particularly added sugars, adjusting total fat intake according to the severity and underlying cause of the TG elevation, and limiting or abstaining from alcohol intake [8].

The NLA recommends increasing protein intake, especially plant-based protein (by 3–5% of TDE), to reduce LDL-C and TG [8]. It highlights the potential added benefit of combining several nutrition interventions aimed at LDL-C-lowering, i.e., replacing 5% of energy from SFA with UFA; consuming 7.5 g/d viscous soluble fiber, 2 g/d plant sterols/stanols, and 30 g/d plant-based protein (as a replacement for animal-based protein and/or carbohydrates); and losing 5% of body weight, if excess adiposity is present [8]. The combination of these interventions is expected to have an LDL-C-lowering effect of 22–37%. The portfolio approach, which combines a low-SFA diet with plant sterols, viscous soluble fiber, soy protein, and almonds, has been shown to yield an LDL-C reduction in controlled-feeding situations of ~30% and in free-living situations of ~12–15% [8,35,36].

It is important to recognize that increasing intake of plant-based protein does not necessarily mean that one should replace all animal-based protein foods. A healthy dietary pattern can include animal sources of protein, including lean cuts of red meat, poultry, dairy, and eggs, without adversely affecting lipoprotein lipid levels or other cardiometabolic risk factors (as described in more detail below) [37,38,39,40,41,42,43]. The main targets for reduction and possible replacement with plant-based protein foods are processed meats, which tend to be higher in SFA and sodium, as well as refined starches and added sugars. The remainder of this article provides insights from RCTs examining the lipoprotein lipid effects of dietary proteins to aid clinicians in the application of protein recommendations from dietary guidance for the general population and for the management of dyslipidemias.

## 5. Effects of Quantity of Dietary Protein on Lipoprotein Lipids

It is widely presumed, based largely on observational evidence, that the effects of total dietary protein on lipoprotein lipids and ASCVD are neutral [44,45,46,47]. However, confounding by differences in total energy intake and substitution effects complicate the interpretation of those data. For example, when total calories are held constant, an increase in one macronutrient (e.g., protein) requires a concurrent decrease in at least one other macronutrient (e.g., carbohydrate or fat); thus, observational evidence is difficult to interpret, and feeding trials are needed to accurately assess the effects of shifts in macronutrient types and subtypes on lipoprotein lipid concentrations [48]. For this reason, we undertook this narrative review focusing on data from RCTs to discuss and summarize the effects on lipids and lipoproteins of substituting proteins for other types of proteins and for other macronutrients.

The Optimal Macronutrient Intake Trial to Prevent Heart Disease (OmniHeart) was an important trial that investigated the effects of macronutrient substitution on the cardiometabolic risk factor profile [49]. It was undertaken to examine the effects on blood pressure and lipoprotein lipids of varying amounts of carbohydrates, UFA, and protein in a “heart-healthy” diet that maintained body weight [49]. OmniHeart was a randomized, three-period, crossover, controlled-feeding (all food provided) study that compared the participants’ (n = 164 adults with prehypertension or stage 1 hypertension) baseline habitual dietary intake (an average American diet) to three variations of the Dietary Approaches to Stop Hypertension (DASH) diet followed for 6 weeks: (1) a higher-carbohydrate DASH diet: 58% of TDE from carbohydrate, 27% from fat, and 15% from protein; (2) a higher-protein DASH diet: 10% of TDE from carbohydrate replaced with protein (~50% plant-based); and (3) a higher-UFA DASH diet: 10% of TDE from carbohydrate replaced with UFA (8% from MUFA and 2% from PUFA). All three DASH diet variations were low in SFA (6% of TDE). Compared to the habitual diet, all of the DASH diet variations significantly lowered fasting total cholesterol, LDL-C, and non-HDL-C concentrations. For both non-HDL-C and TG, the higher-protein and higher-UFA diets produced larger reductions compared to the reductions with the higher-carbohydrate diet (Table 1) [49].

In a randomized, double-blind, controlled-feeding, crossover trial, Maki et al. essentially combined the two most effective interventions from the OmniHeart study, examining the effects on fasting lipoprotein lipids of replacing 16% of energy from refined starches and added sugars with 8% of energy from egg protein and UFA for 3 weeks in participants with overweight or obesity and elevated TG (n = 25) [40]. Energy content was held constant during each diet condition, and all food was provided. The carbohydrate, fat, and protein contents of the diets were approximately 42%, 35%, and 23% of TDE, respectively, in the egg protein + UFA diet condition, and 58%, 27%, and 15% of TDE, respectively, in the refined carbohydrate diet condition. SFA was approximately 7% of TDE in both diet conditions. Compared to the participants’ habitual diets at baseline, both the egg protein + UFA and the refined carbohydrate diet conditions, respectively, significantly (*p* < 0.05) reduced concentrations of total cholesterol (−12.3% and −8.9%), LDL-C (−9.4% and −9.7%), non-HDL-C (−13.3% and −10.4%), HDL-C (−6.4% and −10.2%), and Apo B (−11.6% and −6.4%); however, compared to the refined carbohydrate diet condition, the egg protein + UFA diet also significantly (*p* < 0.05) reduced TG (−18.5% vs. −2.5%) and VLDL-C levels (−18.6% vs. −3.6%) and significantly (*p* < 0.05) increased LDL particle size (0.12 nm vs. −0.15 nm) [40].

A meta-analysis published by Schwingshackl and Hoffmann in 2013 of 15 RCTs comparing the effects of low-fat diets (≤30% of TDE) containing either higher protein (≥25% of TDE) or lower protein (≤20% of TDE), followed for a minimum of 1 year, indicated neutral effects on several cardiovascular risk markers, including total cholesterol (weighted mean difference [WMD] −2.51 mg/dL [−0.06 mmol/L]; *p* = 0.35), LDL-C (WMD 1.58 mg/dL [0.04 mmol/L]; *p* = 0.66), HDL-C (WMD 0.90 mg/dL [0.02 mmol/L]; *p* = 0.08), and TG (WMD −2.87 mg/dL [−0.03 mmol/L]; *p* = 0.49) [50]. However, the authors acknowledged that their results were in contrast to those reported previously by Santesso et al., demonstrating significant increases in HDL-C (21 RCTs; standardized mean difference [SMD] 0.25; *p* = 0.007) and TG (24 RCTs; SMD −0.51; *p* = 0.0002) with higher-protein diets (median 27% of TDE) vs. lower-protein diets (median 18% of TDE) [51]. A potential reason for this discrepancy was the difference in follow-up duration; the majority of the RCTs in the Santesso meta-analysis measured outcomes at <6 months.

The results of a recent systematic review and standard meta-analysis (i.e., combining results from multiple studies that compared the interventions directly) of 56 RCTs that compared consumption of higher-protein diets (>20% of TDE from protein) to lower-protein diets by Yao et al. in studies ranging from 4 to 238 weeks showed a significant reduction in total cholesterol, but not in other lipoprotein lipids [52]. However, the results of a network meta-analysis of these data (i.e., combining results from studies that were connected by common comparators) showed that, compared to lower-protein diets (11–20% of TDE), higher-protein diets favorably reduced total cholesterol, LDL-C, and TG [52]. Like other investigators before them, the authors acknowledged that the overall macronutrient composition of the diet is an important factor, noting that, in their analysis, high-protein, high-carbohydrate (>45%), low-fat (<30%) diets had the greatest effects on total cholesterol and LDL-C lowering, whereas high-protein, moderate-carbohydrate (25–45%), low-fat diets were ranked as the best intervention for reducing TG. Yao et al. also conducted a standard meta-analysis of plant-protein-rich vs. animal-protein-rich diets and reported significant improvements in the lipoprotein lipid profile with plant protein. These results are discussed in greater detail in the next section.

Taken together, these results indicate that higher intakes of dietary protein result in lower levels of atherogenic lipoprotein lipids (LDL-C and non-HDL-C) and Apo B. Moreover, they demonstrate that these effects are mediated in part by substitution for other macronutrients, especially dietary carbohydrates (particularly refined starches and added sugars) and SFA.

## 6. Effects of Source of Dietary Protein on Lipoprotein Lipids

Beyond the effects of the total quantity of dietary protein consumed, evidence indicates that, compared to animal-based proteins, consumption of plant-based proteins significantly reduces concentrations of atherogenic lipoprotein lipids and Apo B [52,53,54,55,56,57,58,59,60]. Again, this is likely due, in part, to substitution effects. A meta-analysis of 112 RCTs (n = 5774 participants), including 34 trials with healthy participants, 51 trials with participants who had hyperlipidemia, and the remaining trials with participants who had other conditions, such as kidney disease, overweight/obesity, type 2 diabetes mellitus, and hypertension, compared the effect on atherogenic lipoprotein lipids and Apo B of 1–2 servings of plant-based proteins for at least 3 weeks as a substitution for animal-based proteins (meat, dairy, and/or egg alternatives) [55]. The substitution of plant-based proteins significantly decreased LDL-C by 6.19 mg/dL (0.16 mmol/L), non-HDL-C by 6.96 mg/dL (0.18 mmol/L), and Apo B by 5 mg/dL (0.05 g/L) (with moderate certainty of evidence), reflecting 3–4% reductions from baseline [55].

Another meta-analysis of 32 studies (n = 1562 participants) that enrolled participants with hypercholesterolemia produced similar results [53]. Plant-based proteins, compared to animal-based proteins, in an isocaloric diet decreased total cholesterol and LDL-C each by 7.35 mg/dL (0.19 mmol/L) and TG by 6.20 mg/dL (0.07 mmol/L); HDL-C was significantly increased by 1.16 mg/dL (0.03 mmol/L) [53]. Likewise, in the meta-analysis by Yao et al. mentioned previously, plant-protein-rich diets, compared to animal-protein-rich diets, significantly (*p* < 0.05) reduced total cholesterol (74 trials; mean difference [MD] −4.64 mg/dL [−0.12 mmol/L]), LDL-C (74 trials; MD −4.25 mg/dL [−0.11 mmol/L]), and TG (70 trials; MD −4.43 mg/dL [−0.05 mmol/L]); and significantly (*p* < 0.05) increased HDL-C (75 trials; MD 1.16 mg/dL [0.03 mmol/L]) [52]. Taken together, these results indicate that plant-based proteins typically have a more favorable effect on lipoprotein lipid concentrations than animal-based proteins.

## 7. Effects of Type of Animal Protein Food or Plant Protein Food on Lipoprotein Lipids

Dietary guidelines usually recommend increasing consumption of plant-based protein foods and decreasing consumption of animal-based protein foods [8,10] in accordance with the RCT data presented above, as well as observational evidence indicating that higher intakes of certain plant-based foods (e.g., whole grains, nuts, seeds, legumes) are associated with lower risks of cardiometabolic diseases and mortality. These associations, if causal, may be partly due to effects on lipoprotein lipids, but also related to effects on other risk determinants, such as biomarkers of inflammation and oxidative stress [60,61,62,63,64]. However, it should be recognized that within the categories of plant proteins and animal proteins are a variety of food sources that not only vary in their amino acid compositions but also in other components with the potential to influence disease risk, such as vitamins, minerals, and phytochemicals [42]. Generally, animal-source foods supply higher amounts of essential amino acids and bioavailable nutrients, such as vitamin B12, iron, zinc, calcium, choline, potassium, and vitamin D, whereas plant-source foods typically have lower amounts of essential amino acids and mineral bioavailability, but are higher in other components, such as fiber, magnesium, folate, pre- and probiotics, and phytochemicals, e.g., polyphenols and plant sterols. Protein foods may also differ in their level of processing (e.g., processed and unprocessed meats, protein isolates). A detailed discussion of lipoprotein lipid effects according to the amount a protein food is processed is beyond the scope of this article.

A systematic review of RCTs that aimed to shift dietary patterns and partially replace animal-based protein foods with plant-based protein foods concluded that this shift improved serum total cholesterol and LDL-C [65]. The three RCTs evaluated were the Effects of Plant and Animal Proteins on Biomarkers of Colorectal Cancer and Type 2 Diabetes in Healthy Adults (ScenoProt) trial, the Study with Appetizing Plant-Food, Meat Eating Alternatives Trial (SWAP-MEAT), and the Replacing Meat with Alternative Plant-Based Products (RE-MAP) trial. The ScenoProt trial is notable because it used a whole-food approach rather than simply swapping animal meat with plant-based meat alternative products. The three diet conditions consumed for 12 weeks were: “Animal” providing 70% animal protein/30% plant protein; “50/50” providing 50% animal protein/50% plant protein; and “Plant” providing 30% animal protein/70% plant protein [66]. In the 50/50 and Plant diets, animal-based protein sources were partly replaced with both new and traditional plant protein sources based on peas, beans, faba (fava) beans, oats, nuts, seeds, and tofu. All diets included the same amount of fish and eggs. Total cholesterol and LDL-C were significantly lower in the Plant group compared to the Animal group (*p* = 0.003 for both), but there were no significant differences in HDL-C or TG.

A controversial animal protein food category is dairy, with some investigators reporting no adverse effects on lipoprotein lipids and others suggesting that certain dairy products have benefits, possibly mediated by their amino acid profiles [67,68,69,70]. A meta-analysis conducted by Zhou et al. evaluated the effects on cardiovascular risk factors in RCTs of individuals with cardiometabolic disease of four types of “high-quality protein” (defined as containing all nine essential amino acids in adequate amounts and proportions, not hydrolysates), including soy protein, milk protein, as well as whey and casein (both milk-derived proteins), compared to control groups without the intervention protein [71]. Both total cholesterol and LDL-C were significantly decreased (−6.96 mg/dL [0.18 mmol/L] and −6.19 mg/dL [0.16 mmol/L], respectively) with soy protein (21 RCTs). Only total cholesterol was significantly decreased (−10.44 mg/dL [−0.27 mmol/L]) with milk protein (2 RCTs), and total cholesterol (−6.96 mg/dL [−0.18 mmol/L]), LDL-C (−3.48 mg/dL [−0.09 mmol/L]), and TG (−8.86 mg/dL [−0.10 mmol/L]) were significantly decreased with whey protein (19–21 RCTs). In contrast, the meta-analysis of three RCTs of casein showed no significant effect on lipoprotein lipids. In the pooled analysis of the high-quality proteins, compared to controls without the intervention protein, among participants with overweight/obesity, total cholesterol (−5.41 mg/dL [–0.14 mmol/L]), LDL-C (−4.64 mg/dL [−0.12, mmol/L]), and TG (−7.97 mg/dL [−0.09 mmol/L]) were each significantly decreased, and HDL-C was significantly increased (0.77 mg/dL [0.02 mmol/L]).

Results from a meta-analysis of 21 RCTs conducted by Prokopidis et al. indicated that whey protein consumed for ≥4 weeks significantly reduced total cholesterol vs. “placebo” (e.g., maltodextrin) or vs. a carbohydrate-based control (MD −6.35 mg/dL [0.16 mmol/L]; *p* = 0.007), but had no significant effects on LDL-C, HDL-C, or TG in the main analysis [69]. However, subgroup analyses suggested larger benefits on the lipoprotein profile in healthy, overweight/obese adults aged <50 years, particularly when combined with exercise. In contrast, a meta-analysis of 20 RCTs conducted by Gataa et al., which did not limit the control group to either a carbohydrate-based control or placebo, demonstrated a significant reduction in TG (WMD −12.21 mg/dL [−0.14 mmol/L]; *p* = 0.003) and a significant increase in HDL-C (WMD 2.59 mg/dL [0.07 mmol/L]; *p* = 0.001), but no significant effects on total cholesterol or LDL-C in the main analysis [68]. However, in subgroup analyses, higher whey dosages (>40 g/d, when the range of dosage was 2–75 g/d) and longer trial durations (>12 weeks, when the range of trial durations was 4–36 weeks) were associated with reductions in total cholesterol and LDL-C. Results from meta-analyses of RCTs examining casein or casein hydrolysate protein reported no significant effect on lipoprotein lipids, including total cholesterol, LDL-C, HDL-C, and TG [71,72].

**Figure 2:**
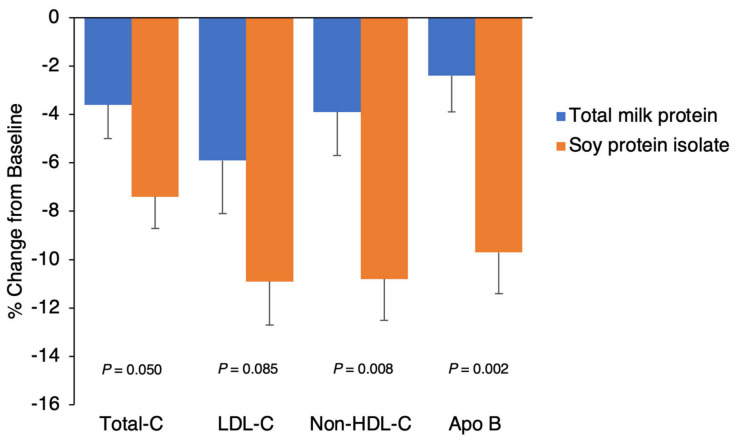
Percentage changes (mean ± SD) from baseline fasting plasma lipoprotein lipids and Apo B after 25 g/d total milk protein or soy protein isolate consumed for 4 weeks by participants with moderate hypercholesterolemia (n = 65) [41]. *p* values are for differences between treatments. Abbreviations: Apo, apolipoprotein; LDL-C, low-density lipoprotein cholesterol; Non-HDL-C, non-high-density lipoprotein cholesterol; Total-C, total cholesterol.

An illustrative example of results from a randomized, controlled, parallel-arm trial comparing the lipoprotein lipid effects of milk protein and soy protein by Maki et al. is shown in Figure 2 [41]. This trial examined the effects on fasting lipoprotein lipid concentrations in participants with hypercholesterolemia who consumed either 25 g/d of a partially proteolytically hydrolyzed soy protein or 25 g/d total milk protein after a 4-week lead-in with a Therapeutic Lifestyle Changes diet [41]. Compared with baseline, both plant and animal protein sources reduced atherogenic lipoprotein lipid and Apo B concentrations, but the responses to soy protein were larger [41]. Compared to baseline, total cholesterol was reduced by 7.4% and 3.6%, LDL-C by 10.9% and 5.9%, non-HDL-C by 10.8% and 3.9% (all at least *p* < 0.05 vs. baseline), and Apo B by 9.7% and 2.4% (significant only for soy protein, *p* < 0.001), with soy and milk proteins, respectively. When comparing the effects of soy protein to milk protein, there were significantly greater reductions in total cholesterol, non-HDL-C, and Apo B (all *p* ≤ 0.05 for between-group differences) and a near-significant greater reduction in LDL-C (*p* = 0.085) with soy protein. Compared to baseline, there was a non-significant decrease in TG (change = −6.8%) with soy protein and a non-significant increase (6.6%) with milk protein (*p* < 0.05 for between group differences), whereas there was a non-significant increase in HDL-C (4.0%) with soy protein and a non-significant decrease in HDL-C (change = −3.3%) with milk protein, compared to baseline (*p* < 0.05 for between group differences). The investigators had hypothesized that soy protein would lower LDL-C and non-HDL-C to a larger degree than milk protein by increasing bile acid excretion [41]. Although the results showed the expected differences in LDL-C and non-HDL-C concentrations, soy protein did not increase excretion of bile acids or neutral sterols compared with milk protein, indicating that other mechanisms account for the differential effects on lipoprotein lipid levels.

Another animal protein food category for which there is substantial debate regarding its healthfulness is red meat. Guideline recommendations from various organizations typically emphasize consuming fish and poultry and limiting red meat (both processed and unprocessed) [11,73,74], based largely on observational evidence suggesting that intake of red meat is associated with increased risks for cancers, type 2 diabetes mellitus, ASCVD, and all-cause mortality [75,76,77,78,79,80]. Processed and unprocessed red meats contain higher amounts of heme iron, trimethylamines (which form trimethylamine N-oxide when gut bacteria metabolize choline, betaine, and L-carnitine), and SFA, which have each been suggested to potentially adversely impact ASCVD risk [81,82,83]. Conversely, fatty fish contain higher amounts of long-chain omega-3 PUFA, including eicosapentaenoic acid and docosahexaenoic acid, which have been associated with reduced risk for ASCVD [84,85]. However, relationships in observational evidence are potentially confounded by the fact that there are other lifestyle differences between individuals who consume more vs. less red meat, such as differences in their consumption of fruits, vegetables, whole grains, fiber, alcohol, SFA, and added sugars, as well as differences in physical activity, smoking history, and body mass index [39].

RCTs comparing red meat to white meat (fish or poultry) to assess their effects on fasting lipoprotein lipid levels have generally failed to indicate differential effects [37,39,86,87,88]. In a crossover RCT of healthy men and women, Bergeron et al. examined the effects on atherogenic lipid and lipoprotein concentrations of consuming diets high in red meat, white meat (poultry), or nonmeat (plant) protein sources (~12% of TDE) on a background diet of either high SFA (~14% of TDE) or low SFA (~7% of TDE) for 4 weeks each [88]. Differences in SFA content between the high- and low-SFA arms were achieved largely with high-fat dairy products and butter. Results demonstrated that LDL-C and Apo B were higher with both red and white meat compared with nonmeat diet conditions, and that this was independent of the SFA content (Table 2) [88]. Furthermore, there were no differences in LDL-C and Apo B concentrations between the red meat vs. white meat diet conditions.

The results of a systematic review and meta-analysis of data from 20 RCTs that evaluated the effects of minimally processed or unprocessed beef intake on cardiovascular risk factors in adults (healthy or with metabolic syndrome or type 2 diabetes mellitus) demonstrated that daily beef intake (~2 servings/d, 161 g/d), compared with diets containing less or no beef, did not significantly affect total cholesterol, non-HDL-C, VLDL-C, HDL-C, Apo B, or Apo A1, but slightly increased LDL-C (~2.7 mg/dL [0.07 mmol/L]; *p* = 0.03) [39]. However, the effect on LDL-C was reduced to non-significance when one influential study, which included a very-low-calorie diet/weight loss lead-in and used the LDL-C value prior to the lead-in as the baseline, was removed from the analysis. Since that meta-analysis, Guzman et al. conducted a randomized, crossover study where 24 adults with overweight or obesity and prediabetes incorporated unprocessed beef or poultry (6–7 oz/d) into their habitual diets for 4-week intervention periods [37]. Results showed no differences in fasting total cholesterol, LDL-C, non-HDL-C, HDL-C, or TG between treatments [37]. Earlier investigations similarly showed that free-living participants with hypercholesterolemia following a National Cholesterol Education Program Step 1 diet containing lean red meat (beef, pork, or lamb) or white meat (poultry or fish) for 36 weeks had similar reductions in total cholesterol and LDL-C and increased HDL-C levels [86,87].

Overall, results from these RCTs and meta-analyses support differential effects of protein source and type on lipoprotein lipid levels. Plant-based proteins generally produced larger effects on lipoprotein lipid concentrations than proteins from animal sources. However, some differential responses were observed between protein fractions from a single source, such as significant reductions in total cholesterol and LDL-C with whey protein that were not observed with casein. Table 3 summarizes the results from selected meta-analyses where the protein effect can be more clearly isolated from confounding factors, such as differences between conditions in fatty acids, cholesterol, fiber, food matrix, and other bioactives.

Some of the uncertainties in the literature relating to effects of different types of protein on lipoprotein lipids may stem from differences in the food matrix, degree of processing, or comparator group. For example, plant-based food matrices with relatively little processing likely contain many nutrients and bioactives within a cellular structure that must be broken down before the nutrients can be accessed. Industrial processing, cooking, and even chewing can alter the bioavailability of these nutrients, which may influence the lipoprotein lipid response. Interpretation of results from studies on dairy foods can be particularly challenging as various processes such as heat treatments, homogenization, and fermentation have all been shown to change the micro- and macrostructures of the dairy matrix, which may influence the impacts on lipoprotein lipids [89].

## 8. Proposed Mechanisms for the Lipid-Lowering Effects of Proteins

The mechanisms whereby increasing total dietary protein intake affects lipoprotein lipid concentrations are not clearly understood and have not been validated in clinical settings. These proposed mechanisms are summarized in Table 4. One proposed mechanism is substitution effects, which are, as mentioned earlier, when total energy intake is held constant, increasing dietary protein intake is associated with a reduction in the intake of other nutrients with potential adverse effects on lipoprotein lipids, e.g., refined carbohydrates and SFA. These substitution effects are also likely at least partly responsible for the improvements in lipoprotein lipid concentrations with the replacement of some animal-based protein foods, which may also act as a vehicle for other dietary components that raise atherogenic lipoprotein lipids (e.g., SFA, cholesterol), with plant-based protein foods, which may act as a vehicle for other dietary components that lower atherogenic lipoprotein lipids (e.g., viscous soluble fibers, phytosterols, pre- and probiotics) [52,55,58,90,91]. Another possible mechanism is that, as the most thermogenic macronutrient, protein increases energy expenditure and satiety and promotes modest weight loss, which may, in turn, lead to improvements in several biomarkers of cardiometabolic health, including reducing concentrations of atherogenic lipoprotein lipids [20,92].

Other mechanistic pathways for the reduction in atherogenic lipoprotein lipids with increased dietary protein intake, which have been studied largely in animal models and cell systems, involve potential effects on hepatic gene expression, including inhibition of genes necessary for TG synthesis and VLDL particle secretion, and upregulation of genes involved in hepatic fatty acid uptake and oxidation and removal of cholesterol from the circulation, but evidence in humans is lacking [52,93,94,95]. In general, animal-based proteins are higher in essential amino acids, including the branched-chain amino acids (BCAA; leucine, isoleucine, valine), whereas plant-based proteins are higher in the nonessential amino acids, such as arginine and glycine [52,94,95,96]. Increased circulating levels of BCAA and related metabolites are a metabolic hallmark of ASCVD [97,98]. They influence lipid metabolism by stimulating the mechanistic target of rapamycin (mTOR), which impacts the expression of lipid genes, leading to elevated circulating free fatty acids, TG, and LDL-C levels [97,98,99]. However, results from some studies have suggested that BCAA may have cardioprotective effects under certain conditions [98]. Furthermore, whey protein, which is rich in BCAA, leads to improved lipoprotein lipid levels in humans [68,69]. The complexity of this multifaceted relationship warrants further investigation. Dietary protein, particularly plant-based protein, has also been suggested to enhance bile acid excretion; however, to date, this has not been demonstrated in humans [100]. As discussed previously, in a study in which participants with hypercholesterolemia consumed total milk protein or the insoluble fraction of soy protein, both protein sources reduced atherogenic lipoprotein lipids but neither increased bile acid excretion [41].

Food-derived polypeptides, made up of two or more amino acids linked by peptide bonds produced by the enzymatic breakdown of proteins from marine, plant, and animal sources, are emerging candidates for the management of hyperlipidemia [101,102,103,104]. Several antihyperlipidemic mechanisms of peptides have been demonstrated in cells and animal models, including: (1) competitive inhibition of 3-hydroxy-3-methylglutaryl coenzyme A (HMG CoA) reductase activity by mimicking the 3-dimensional structure of HMG CoA and binding to its active site to prevent cholesterol synthesis and reduce circulating cholesterol; (2) promotion of cholesterol excretion by mimicking the structure of bile acids and binding to receptors on liver cells to reduce bile acid synthesis, block enterohepatic circulation of bile acids, and stimulate bile acid secretion; and (3) regulation of lipoprotein metabolism by binding to receptors on the cell surface of the liver to inhibit fatty acid synthesis and regulate Apo B100 expression, as well as the activity of other lipid metabolism-related genes, resulting in reduced VLDL synthesis and increased LDL receptor expression [101,102]. However, peptides are easily decomposed, have low bioavailability, and their metabolism in the human body is complex. Strategies to deliver bioactive peptides through functional food development need to be investigated in clinical trials in humans before inferences can be drawn regarding potential clinical usefulness [105].

## 9. Conclusions and Future Research

In conclusion, dietary recommendations for the general public, as well as for the management of dyslipidemias, emphasize the importance of dietary protein as part of a healthy dietary pattern composed of whole, unprocessed, or minimally processed foods, with high intakes of fiber-rich components, such as fruits, vegetables, whole grains, nuts, seeds, and legumes, as well as healthy fats. Both animal-based and plant-based proteins improve atherogenic lipoprotein lipid (LDL-C and non-HDL-C) concentrations, in part by replacing the intakes of foods high in dietary components with adverse effects, such as refined carbohydrates (starches and added sugars), SFA, and cholesterol, but also through other mechanisms that are incompletely understood. Plant-based proteins typically have more favorable effects on lipoprotein lipid concentrations than animal-based proteins, again, likely due in part to substitution of refined carbohydrates or SFA, but possibly also due to food matrix and/or processing effects. However, within the broad categories of animal proteins and plant proteins are various food types that differ not only in their amino acid composition but also in their contents of other nutrients and bioactive compounds, which leads to varying responses according to protein food type. Because this is a narrative review, no formal systematic review protocol was applied, and part of the evidence discussed is derived from heterogeneous intervention studies that differed in protein source, dose, comparator, food matrix, duration, and study population. Accordingly, these conclusions should be interpreted within the scope of a narrative approach and with recognition of the variability in the studies that contribute to the evidence base. Additional research is warranted to further elucidate the optimal quantities and food sources of dietary proteins, as well as mechanisms for the effects of dietary proteins on lipoprotein lipid metabolism.

## Figures and Tables

**Table 1:** Lipoprotein lipid concentrations at baseline (habitual diet) and after 6-week interventions with higher-CHO, higher-PRO, and higher-UFA diets ^1^ in OmniHeart (n = 164 adults with prehypertension or stage 1 hypertension) [49].

Lipoprotein Lipid ^2^	Baseline	Higher CHO	Higher PRO	Higher UFA
	Mean (SD) ^3^	Change from Baseline, Mean (95% CI) ^4−8^		
Total-C (mg/dL)	204 (35.7)	−12.4 (−15.7, −9.1)	−19.9 (−23.5, −16.4)	−15.4 (−19.1, −11.8)
LDL-C (mg/dL)	129 (32.4)	−11.6 (−14.6, −8.6)	−14.2 (−17.5, −10.9)	−13.1 (−16.4, −9.8)
Non-HDL-C (mg/dL)	154 (36.8)	−11.0 (−14.2, −7.8)	−17.3 (−20.8, −13.8)	−15.1 (−18.6, −11.6)
HDL-C (mg/dL)	50.0 (16.1)	−1.4 (−2.5, −0.3)	−2.6 (−3.6, −1.6)	−0.3 (−1.3, 0.7)
TG (mg/dL)	102 (75–159)	0.1 (−8.6, 8.8)	−16.4 (−25.5, −7.3)	−9.3 (−17.5, −1.2)

^1^ Higher CHO = % TDE from CHO (58%), PRO (15%), and fat (27%: 6% SFA, 13% MUFA, 8% PUFA); Higher PRO = % TDE from CHO (48%), PRO (25%), and fat (27%: 6% SFA, 13% MUFA, 8% PUFA); Higher UFA = % TDE from CHO (48%), PRO (15%), and fat (37%: 6% SFA, 21% MUFA, 10% PUFA). ^2^ To convert cholesterol in mg/dL to mmol/L, multiply by 0.0259; to convert TG in mg/dL to mmol/L, multiply by 0.0113. ^3^ For TG, baseline values are median (IQRL). ^4^ Between diet differences for Total-C: Higher PRO vs. Higher CHO, *p* < 0.001; Higher UFA vs. Higher CHO, *p* = 0.04; Higher PRO vs. Higher UFA, *p* < 0.001. ^5^ Between diet differences for LDL-C: Higher PRO vs. Higher CHO, *p* = 0.01; Higher UFA vs. Higher CHO, *p* = 0.22; Higher PRO vs. Higher UFA, *p* = 0.24. ^6^ Between diet differences for Non-HDL-C: Higher PRO vs. Higher CHO, *p* < 0.001; Higher UFA vs. Higher CHO, *p* = 0.002; Higher PRO vs. Higher UFA, *p* = 0.054. ^7^ Between diet differences for HDL-C: Higher PRO vs. Higher CHO, *p* = 0.02; Higher UFA vs. Higher CHO, *p* = 0.03; Higher PRO vs. Higher UFA, *p* < 0.001. ^8^ Between diet differences for TG: Higher PRO vs. Higher CHO, *p* < 0.001; Higher UFA vs. Higher CHO, *p* = 0.02; Higher PRO vs. Higher UFA, *p* = 0.03. Abbreviations: CHO, carbohydrate; HDL-C, high-density lipoprotein cholesterol; IQRL, interquartile range limits; LDL-C, low-density lipoprotein cholesterol; MUFA, monounsaturated fatty acids; non-HDL-C, non-high-density lipoprotein cholesterol; OmniHeart, Optimal Macronutrient Intake Trial to Prevent Heart Disease; PRO, protein; PUFA, polyunsaturated fatty acids; SFA, saturated fatty acids; TDE, total daily energy; TG, triglycerides; Total-C, total cholesterol; UFA, unsaturated fatty acids.

**Table 2:** Lipoprotein lipid and Apo B concentrations in healthy men and women after 4 weeks of following diets varying in type of dietary protein and SFA content [88].

Lipoprotein Lipid ^1^	High SFA (n = 62)			Low-SFA (n = 51)		
	Red Meat	White Meat	Nonmeat	Red Meat	White Meat	Nonmeat
Total-C ^2^ (mg/dL)	171 (36.0)	170 (32.1)	163 (32.1)	159 (30.2)	160 (30.9)	154 (30.9)
LDL-C ^3^ (mg/dL)	102 (30.9)	101 (27.8)	95.1 (27.1)	90.9 (22.8)	92.0 (25.1)	85.9 (25.1)
Non-HDL-C ^4^ (mg/dL)	119 (36.0)	118 (32.9)	113 (32.9)	105 (27.1)	106 (27.8)	100 (29.0)
Apo B ^5^ (mg/dL)	73.0 (23.0)	74.0 (22.0)	70.0 (21.0)	67.0 (18.0)	67.0 (19.0)	63.0 (18.0)

^1^ For cholesterol, to convert to mmol/L, multiply by 0.0259. For Apo B, to convert to g/L, multiply by 0.01. ^2^ Difference between proteins, *p* < 0.0001; difference between SFAs, *p* = 0.0002; *p* interaction = 0.69. ^3^ Difference between proteins, *p* < 0.0001; difference between SFAs, *p* = 0.0003; *p* interaction = 0.63. ^4^ Difference between proteins, *p* < 0.0001; difference between SFAs, *p* = 0.0003; *p* interaction = 0.83. ^5^ Difference between proteins, *p* < 0.0001; difference between SFAs, *p* = 0.0002; *p* interaction = 0.99. Abbreviations: Apo, apolipoprotein; LDL-C, low-density lipoprotein cholesterol; Non-HDL-C, non-high-density lipoprotein cholesterol; SFA, saturated fatty acids; Total-C, total cholesterol.

**Table 3:** Summary table of the results of select meta-analyses evaluating the effect of different sources and types of protein on lipoprotein lipid levels.

	No. Studies/ No. Participants	Mean Difference ^1^ (95% CI)	*p* Value	Heterogeneity I^2^	Evidence Strength	Summary Conclusions
**Plant vs. animal protein [55]**						
LDL-C (mg/dL)	108/5582	−6.19 (−7.73, −4.64)	<0.0001	55%	Moderate	Substituting plant protein for animal protein decreases LDL-C, non-HDL-C, and Apo B
Non-HDL-C (mg/dL)	102/5401	−6.96 (−8.51, −5.41)	<0.0001	52%	Moderate	
Apo B (mg/dL)	37/1506	−5.0 (−6.0, −3.0)	<0.0001	30%	Moderate	
**Soy protein [57]**						
Total-C (mg/dL)	52/1691	−6.41 (−9.30, −3.52)	<0.001	74%	NR	Soy protein lowers total-C and LDL-C
LDL-C (mg/dL)	50/1661	−4.76 (−6.71, −2.80)	<0.0001	55%	NR	
**Whey protein [69]**						
Total-C (mg/dL)	13/627	−6.35 (−10.96, −1.74)	0.007	71%	NR	Whey protein lowers total-C and LDL-C, but has no effect on HDL-C or TG
LDL-C (mg/dL)	12/609	−2.07 (−4.10, −0.03)	0.05	6%	NR	
HDL-C (mg/dL)	13/627	−0.82 (−2.17, 0.53)	0.23	43%	NR	
TG (mg/dL)	13/627	−3.20 (−8.03, 1.62)	0.19	78%	NR	
**Casein protein hydrolysate [72]**						
Total-C (mg/dL)	13/672	−2.71 (−6.56, 1.15)	0.17	0%	NR	Casein protein hydrolysates have no effect on lipoprotein lipid levels
LDL-C (mg/dL)	7/386	−1.54 (−5.80, 3.09)	0.54	0%	NR	
HDL-C (mg/dL)	11/641	−0.39 (−2.32, 1.16)	0.55	0%	NR	
TG (mg/dL)	14/702	−4.43 (−12.40, 4.43)	0.33	0%	NR	

^1^ For cholesterol, to convert to mmol/L, multiply by 0.0259. For TG, to convert to mmol/L, multiply by 0.0113. For Apo B, to convert to g/L, multiply by 0.01. Abbreviations: Apo, apolipoprotein; HDL-C, high-density lipoprotein cholesterol; LDL-C, low-density lipoprotein cholesterol; Non-HDL-C, non-high-density lipoprotein cholesterol; NR, not reported; TG, triglycerides; Total-C, total cholesterol.

**Table 4:** Proposed mechanisms by which dietary protein quantity, source, and type may influence lipoprotein lipids.

Proposed Mechanism ^1^	Concept	Potential Relevance for Lipoprotein Lipids	Caveats to Consider in the Evidence
Substitution effects	Protein replaces refined carbohydrates, added sugars, or SFA	Lower LDL-C, non-HDL-C, or TG depending on nutrient displaced	Most plausible pathway but effects depend on nutrient displaced
Food matrix and accompanying bioactive components	Plant and animal sources differ in dietary fiber, phytosterols, fatty acids, cholesterol, sodium, and processing	Accompanying dietary components can increase or decrease lipoprotein lipids, independent of protein	Difficult to isolate protein-specific effects from other components
Energy balance and body weight	Proteins may increase satiety and thermogenesis to support modest weight loss	Improvements in lipoproteins secondary to weight changes	Potential effects depend on level of energy restriction, adherence, baseline weight, and study duration
Hepatic lipid metabolism	Potential effects of protein on hepatic TG synthesis, VLDL secretion, fatty acid uptake and oxidation	May lead to lower LDL-C, VLDL, and non-HDL-C	Human evidence limited—primarily animal and cell models
BCAA and amino acid profiles	May influence lipid metabolism through mTOR	Potential elevations in free fatty acids, TG, and LDL-C	Human evidence is not consistent (e.g., whey is high in BCAA and lowers LDL-C)
Bile acid metabolism	Proteins may increase bile acid excretion	Potential to reduce circulating cholesterol	Has not been demonstrated in humans
Food-derived peptides	May inhibit HMG-CoA reductase, increase bile acid excretion, and regulate Apo B100 expression	Potential to reduce circulating cholesterol and VLDL synthesis. Possibly increase LDL receptor expression	Peptides generally have low bioavailability in humans and clinical data are lacking

^1^ These pathways are hypothesized and have variable evidentiary support. Abbreviations: Apo, apolipoprotein; BCAA, branched-chain amino acids; HMG-CoA, 3-hydroxy-3-methylglutaryl coenzyme A; LDL-C, low-density lipoprotein cholesterol; mTOR, mechanistic target of rapamycin; non-HDL-C, non-high-density lipoprotein cholesterol; SFA, saturated fatty acids; TG, triglycerides; VLDL, very low-density lipoprotein.

## Data Availability

No new data were created or analyzed in this study.

## Abbreviations

The following abbreviations are used in this manuscript:

Apo	apolipoprotein
ASCVD	atherosclerotic cardiovascular disease
BCAA	branched-chain amino acids
CHO	carbohydrate
DASH	Dietary Approaches to Stop Hypertension
DGA	Dietary Guidelines for Americans
DHA	docosahexaenoic acid
EPA	eicosapentaenoic acid
HDL-C	high-density lipoprotein cholesterol
HMG CoA	3-hydroxy-3-methylglutaryl coenzyme A
LDL	low-density lipoprotein
LDL-C	low-density lipoprotein cholesterol
mTOR	mechanistic target of rapamycin
MUFA	monounsaturated fatty acids
NLA	National Lipid Association
Non-HDL-C	non-high-density lipoprotein cholesterol
OmniHeart	Optimal Macronutrient Intake Trial to Prevent Heart Disease
PRO	protein
PUFA	polyunsaturated fatty acids
RCTs	randomized controlled trials
SFA	saturated fatty acids
TDE	total daily energy
TFA	*trans* fatty acids
TG	triglycerides
Total-C	total cholesterol
UFA	unsaturated fatty acids
VLDL	very low-density lipoprotein
VLDL-C	very low-density lipoprotein cholesterol
WHO	World Health Organization
